# High Lyso-Gb3 Plasma Levels Associated with Decreased miR-29 and miR-200 Urinary Excretion in Young Non-Albuminuric Male Patient with Classic Fabry Disease

**DOI:** 10.1155/2019/4980942

**Published:** 2019-01-10

**Authors:** Sebastián Jaurretche, Germán R. Perez, Graciela Venera

**Affiliations:** ^1^Physiology Department, School of Medicine, Instituto Universitario Italiano de Rosario, Rosario, Argentina; ^2^Los Manantiales, Neurosciences Center, Rosario, Argentina; ^3^School of Biochemical and Pharmaceutical Sciences, National University of Rosario, Rosario, Argentina; ^4^Gammalab Grupo Gamma, Rosario, Argentina; ^5^Research Department, School of Medicine, Instituto Universitario Italiano de Rosario, Rosario, Argentina

## Abstract

Renal involvement is associated with a greater morbidity and mortality in Fabry disease. Pathological albuminuria, the first Fabry nephropathy clinical manifestation, can occur from early childhood, although histological lesions such as tubulo-interstitial fibrosis and glomerulosclerosis are present or may precede the onset of pathological albuminuria. In renal cells, exposure to Lyso-Gb3 is correlated with increased expression of Transforming Growth Factor-*β*eta (TGF-*β*). miR-21, miR-192, and miR-433 that promote fibrosis are activated by TGF-*β*, and miR-29 and miR-200 that suppress fibrosis are inhibited by TGF-*β*. A 23-year-old male was diagnosed with FD. *α*GalA decreased enzyme activity: 0.1 nmol/hour/liter; genotype: [c.317T>G (p.L106R)]; GFR: 104.4 mL/min/m^2^; urinary albumin excretion: 6.00 mg/day; plasma Lyso-Gb3: 124.5 nmol/L. A decrease urinary excretion of miR-29 and miR-200 was found (p <0.005) compared to controls. In addition to its usefulness as a phenotype marker, Lyso-Gb3 has been proposed as an indicator of therapeutic response. We detect an association of high Lyso-Gb3 plasma values with decreased urinary excretion of miRNAs with known antifibrotic effect (miR-29 and miR-200). Although the present work is a case report, it could be hypothesized that one of the harmful Lyso-Gb3 effects could be the miRNAs regulation through changes in TGF-*β* expression.

## 1. Introduction

Fabry disease (FD) is an X-linked *α*-galactosidase-A (*α*GalA) deficiency which results in the failure of glycosphingolipids catabolism, particularly globotriaosylceramide (Gb3) and globotriaosylsphingosine (Lyso-Gb3) [1]. The progressive glycosphingolipids storage in lysosomes of different cells starts in fetal life [1] and leads to deteriorated cellular and tissue function which cause FD clinical manifestations [1].

Nephropathy is a major FD complication and mainly includes proteinuria and progressive decreased glomerular filtration rate (GFR) [1, 2]. Renal involvement is associated with a greater morbidity and mortality when it is present in affected patients both males and females [1, 3]. Over the last years, research has shown that females carrying mutated allele also develop symptoms, demonstrating a wide range of disease severity, from a virtually asymptomatic to more classical phenotype, with cardiac, renal, and cerebrovascular manifestations. This variable expression in females is thought to be influenced by the process of X-chromosome inactivation (Lyon hypothesis) [1]. Males with classical GLA gene mutations develop overt proteinuria and progressive renal impairment by the second-to-fifth decade of life, while in women the renal involvement has been described to be milder and later than in men [1].

Pathological albuminuria, which is long considered the first clinical manifestation of Fabry nephropathy, can occur from early childhood [1, 4], although it is well known that morphological changes, even irreversible, such as tubulointerstitial fibrosis and glomerulosclerosis, are present or may precede the onset of pathological albuminuria [5, 6].

In Fabry nephropathy, glycolipids deposit in glomerular, tubular, and vascular cells leading to glomerulosclerosis, tubular atrophy, interstitial fibrosis, and inflammation [5, 7].

Lyso-Gb3, a deacylated Gb3, increases in plasma of FD patients [1]. The highest Lyso-Gb3 plasma concentrations have been observed in males affected by most severe FD form (“Classic” or “Type I” FD) [8]. The Lyso-Gb3 deleterious effects on podocytes and renal tubular cells have been demonstrated in both animal and human models of Fabry nephropathy [9, 10]. In podocytes, exposure to Lyso-Gb is correlated with increased Transforming Growth Factor-*β*eta (TGF-*β*) and extracellular matrix components expression, both mechanisms associated with renal fibrosis development [9]. In renal tubular cells exposed to Lyso-Gb3, epithelial-mesenchymal transition has been described as another mechanism related to renal fibrosis [10].

TGF-*β* also regulates several microRNAs (miRs; miRNAs) during renal fibrosis, suggesting that they play essential roles in this process. miR-21, miR-192, and miR-433 that promote fibrosis are activated by TGF-*β*, and miR-29 and miR-200 that suppress fibrosis are inhibited by TGF-*β* [11]. miRNAs are small, endogenous, non-coding RNAs that regulate various cellular processes. Several studies suggest the potential role of miRNAs in kidney physiology [11].

We have recently demonstrated that young FD patients present a miRNAs urinary profile excretion indicative of renal fibrosis in correlation with decreased *α*GalA activity, even in non-albuminuric patients [12]. This work includes the study of miRNAs families regulated by TGF-*β*. We have observed that, in comparison with the control group, the relative excretion levels of miR-29 and miR-200 families were decreased, while miR-21, miR-192, and miR-433 were not [12].

The aim of this work is to describe the clinical case of a young FD patient without nephropathy manifestations, high Lyso-Gb3 plasma levels, decreased *α*GalA enzyme activity, and relative urinary excretion levels of miR-29 and miR-200 diminished.

## 2. Case Presentation and Methods

**Figure 1:**
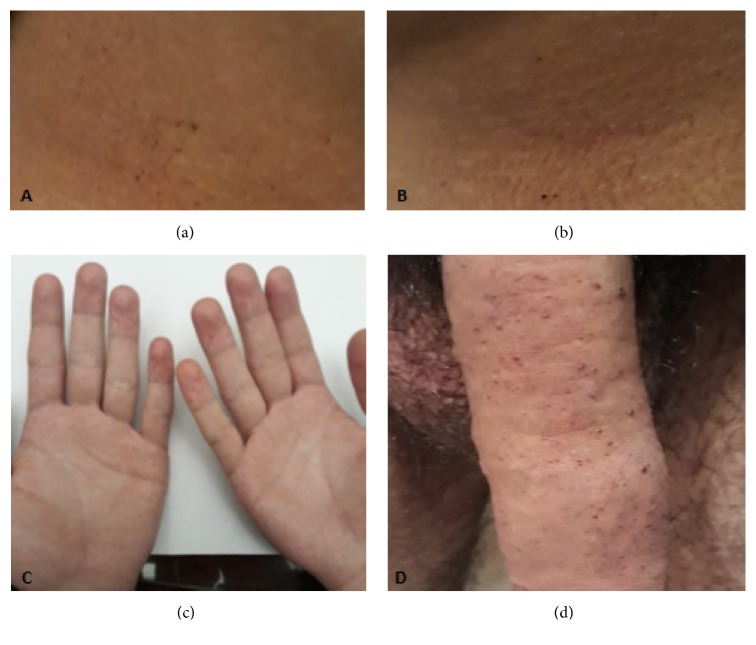
**Typical skin lesions (angiokeratomas) of Fabry disease. **(a) and (b) Angiokeratomas in lower abdomen skin. (c) Angiokeratomas in skin of hands. (d) Angiokeratomas in penile skin.

**Figure 2:**
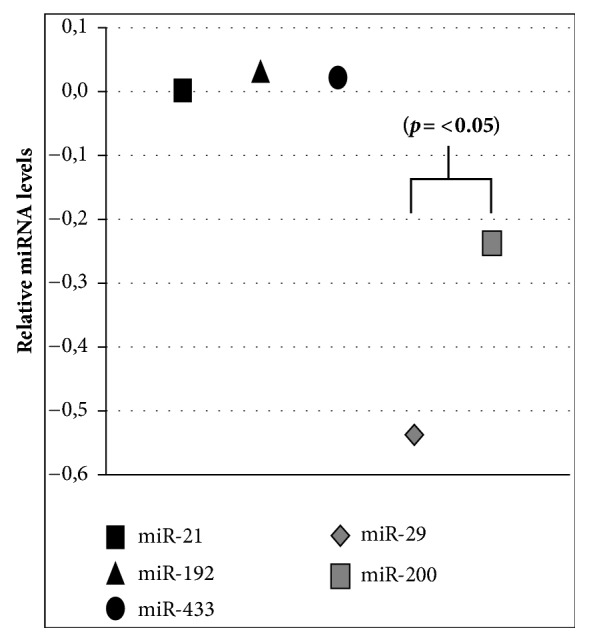
**Relative expression levels of miRNAs in urinary sediment versus controls: **Y axis represents 2^−ΔΔCt^ miRNAs values calculated by Delta-Delta Ct (ΔΔCt) method versus control. Expression was normalized to U6, and data are represented as means ± SEM.

A 23-year-old male who suffered from acroparesthesias, decreased sweating, exercise intolerance, and frequent episodes of diarrhea and abdominal discomfort was diagnosed with FD. Physical examination: angiokeratomas on palms and genitals were found (Figure 1). The *α*GalA test in dried blood spot disclosed decreased enzyme activity, 0.1 nmol/hour/liter (normal > than 4 nmol/hour/liter). A mutation [c.317T>G (p.L106R)] was identified in the GLA gene, by sequential analysis. The laboratory results were unremarkable, with a GFR of 104.4 mL/min/m^2^ estimated by the CKD-EPI equation, 24-hour urinary albumin excretion 6.00 mg/day. A renal ultrasound and DOPPLER echocardiogram were normal. A plasma Lyso-Gb3 value of 124.5 nmol/L was found, determined by tandem mass spectrometry method. To detect the relative excretion urinary levels of miR-21, miR-29, miR-192, miR-200, and miR-433, reverse transcription reaction with a stem-loop primer was used. The resulting cDNA was amplified using a miRNA-specific forward primer and the universal reverse primer [12]. Relative miRNAs expression levels were calculated using the 2^-ΔΔCt^ method as previously described [13] (Figure 2).

After the FD diagnostic confirmation enzyme replacement therapy with agalsidase-beta at a dose of 1 mg/Kg/every other week was indicated.

## 3. Discussion and Conclusion

The mechanisms that lead to renal fibrosis in FD patients are not completely understood. However, it is well known that Fabry nephropathy progresses to renal fibrosis and that fibrotic lesions may precede pathological albuminuria, the first clinical manifestation of renal involvement in affected patients [1, 2, 4, 5].

The origin of excessive plasma accumulation of Lyso-Gb3 in affected patients is not clearly identified. It has been proposed that it comes from the deacylation of abnormally accumulated Gb3 molecules due to the deficit of *α*GalA [9, 10, 14]. Elevated plasma Lyso-Gb3 is a hallmark of classical FD in males although in Fabry females it is relatively lower [15]. In addition to its usefulness as a phenotype marker, Lyso-Gb3 has been proposed as an indicator of therapeutic response. It has been reported that ERT is capable of decreasing or stabilizing plasma Lyso-Gb3 levels, mainly in patients treated with high doses of ERT [15]. Lyso-Gb3 has been associated with some FD clinical manifestations in various organs. In the present report, pain was the most prominent clinical manifestation and also motivated the consultation. In animal models a direct correlation between the administration of Lyso-Gb3 and allodynia has been demonstrated [14]. In certain circuits of sensory neurons, lyso-Gb3 induces an increase in intracellular calcium by a functional upregulation of voltage-dependent Ca^2+^ channels [14].

Gb3 and lyso-Gb3 are involved with various cellular mechanisms implying renal fibrosis [9, 10]. Glycolipids, mainly Gb3 are deposited in all types of kidney cells [5, 6]. The abnormal deposit of Gb3 has been associated with tissue ischemia due to microvascular occlusion and its final result, the renal fibrosis [1].

Sanchez Niño et al. have described that lyso-Gb3 dose- and time-dependently increased the TGF-*β*1expression, extracellular matrix proteins (fibronectin and type IV collagen), and CD74, in human podocytes [9]. Jeon et al. (2015) described that FD renal tubular cells treated with Gb3 and lyso-Gb3 showed a transition from epithelial to mesenchymal cells, a key event in the development of renal fibrosis of any cause [10]. Both groups described that this Gb3 and lyso-Gb3 effect is mediated by TGF-*β*.

We report for the first time that young FD patients with classical GLA gene mutations and mild or absent nephropathy presented a miRNAs urinary excretion profile indicative of renal fibrosis, even in pre-albuminuric stage. This profile was produced by a decrease of miRNAs downregulated by TGF-*β* (miR-29 and miR-200) and significantly correlated with decreased *α*GalA activity [12].

In the presented case, plasma Lyso-Gb3 levels within the mean values of patients with the classic FD were found [8]. We detect an association of high plasma Lyso-Gb3 values with decreased urinary excretion of miRNAs that suppress fibrosis (miR-29 and miR-200). On the other hand, normal values of miRNAs miR-21, miR-192, and miR-433 with recognized profibrotic effect were observed. Although the present work is a case report, it could be assumed that one of harmful Lyso-Gb3 effects could be the miRNAs regulation through changes in TGF-*β* expression.

## Data Availability

The data used to support the findings of this study are restricted by the Ethic Committee of the Instituto Universitario Italiano de Rosario in order to protect patient privacy. Data are available for researchers who meet the criteria for access to confidential data
